# Inhibition of cancer cell epithelial mesenchymal transition by normal fibroblasts via production of 5-methoxytryptophan

**DOI:** 10.18632/oncotarget.9111

**Published:** 2016-04-29

**Authors:** Huei-Hsuan Cheng, Ling-Yun Chu, Li-Yi Chiang, Hua-Ling Chen, Cheng-Chin Kuo, Kenneth K. Wu

**Affiliations:** ^1^ Metabolomic Medicine Research Center, China Medical University Hospital, Taichung, Taiwan; ^2^ Graduate Institute of Clinical Medical Science, China Medical University, Taichung, Taiwan; ^3^ Institute of Cellular and System Medicine, National Health Research Institutes, Zhunan, Taiwan; ^4^ Institute of Biotechnology, National Tsing-Hua University, Hsinchu, Taiwan

**Keywords:** epithelial mesenchymal transition, fibroblasts, lung cancer, 5-methoxytryptophan, tryptophan hydroxylase-1

## Abstract

We reported previously that human fibroblasts release 5-methoxytryptophan (5-MTP) which inhibits cancer cell COX-2 overexpression and suppresses cancer cell migration and metastasis. To determine whether fibroblasts block cancer cell epithelial mesenchymal transition (EMT) via 5-MTP, we evaluated the effect of Hs68 fibroblasts (HsFb) on A549 cancer cell EMT in a two-chamber system. Co-incubation of A549 with HsFb prevented TGF-β1-induced reduction of E-cadherin and increase in Snail and N-cadherin. Transfection of HsFb with tryptophan hydroxylase-1 siRNA, which inhibited tryptophan hydroxylase-1 protein expression and 5-MTP release in HsFb abrogated the effect of HsFb on A549 EMT. Direct addition of pure 5-MTP to cultured A549 cells followed by TGF-β1 prevented TGF-β1-induced reduction of E-cadherin, and elevation of Snail, vimentin and matrix metalloproteinase 9. Administration of 5-MTP to a murine xenograft tumor model reduced vimentin protein expression in the tumor tissues compared to vehicle control which was correlated with reduction of metastasis in the 5-MTP treated mice. Our experimental data suggest that 5-MTP exerted its anti-EMT actions through inhibition of p38 MAPK activation, p65/p50 NF-κB nuclear translocation and transactivation without the involvement of COX-2 or p300 histone acetyltransferase. Our findings indicate that fibroblasts release a tryptophan metabolite, 5-MTP, to reduce cancer cell EMT, migration, invasion and metastasis.

## INTRODUCTION

Fibroblasts are elongated spindle-like cells of mesodermal origin. They express complex surface markers reflecting inherent diversity and heterogeneity. Fibroblasts are widely distributed in many tissues and produce collagen and extracellular matrix (ECM) to support tissue integrity. They respond to injury signals and migrate to the injury sites to carry out repair work [1, 2]. Fibroblasts are recruited by cancer cells to the tumor microenvironment where they play an active role in regulating cancer growth and metastasis [3]. Much attention has focused on cancer-associated fibroblasts (CAF) which are considered to be altered by cancer cells to promote cancer growth [4]. However, recent studies indicate that CAFs are heterogeneous [5] and do not uniformly promote cancer growth. They may suppress cancer progression [6, 7]. On the other hand, several reports indicate that naive normal fibroblasts reduce cancer growth through direct cancer cell contacts [8, 9] or via releasing soluble factors [10]. The mechanisms by which CAFs or normal fibroblasts suppress cancer growth are not well understood. We have identified by comparative metabolomics a tryptophan metabolite i.e. 5-methoxytryptophan (5-MTP, also known as cytoguardin) as a soluble factor released by human fibroblasts, which reduces cyclooxygenase-2 (COX-2) overexpression by blocking p300 histone acetyltransferase (HAT) and NF-κB activation [11, 12]. We previously reported that 5-MTP inhibits A549 cancer cell migration and invasion and retards A549 cancer growth and metastasis in a murine xenograft tumor model possibly through COX-2 suppression [11].

Epithelial mesenchymal transition (EMT) is a crucial cancer cell phenotypic change which enables cancer cells to detach from the epithelial adhesion and migrate to distant sites [13, 14]. Cancer cell EMT is induced by growth factors notably transforming growth factor-β1 (TGF-β1) [15, 16]. TGF-β1 induces cancer cell EMT by promoting expression of Snail, which represses E-cadherin expression and thereby disrupts epithelial junction [17–19]. Snail also enhances the expression of N-cadherin, vimentin and fibronectin which confer mesenchymal phenotypic features. Mesenchymal phenotypic switch is accompanied by production of matrix metalloproteinases (MMPs) which degrade extracellular matrix to facilitate cancer cell migration and invasion [20]. CAFs were reported to regulate cancer cell EMT [21–23]. However, little is known about the influence of normal naive fibroblasts over cancer cell EMT. We postulate that normal fibroblasts control EMT via 5-MTP production. To test this hypothesis, we used Hs68 fibroblasts and A549 lung cancer cells as a cell model. Our results reveal that fibroblasts produce soluble factors to block TGF-β1-induced A549 EMT which was abrogated by silencing tryptophan hydroxylase-1 (TPH-1), a key enzyme in 5-MTP synthesis. Addition of 5-MTP rescues EMT control by inhibiting TGF-β1-induced activation of p38 MAPK signaling pathway and NF-κB transactivation.

## RESULTS

### Fibroblasts release soluble factors to inhibit A549 EMT

**Figure 1:**
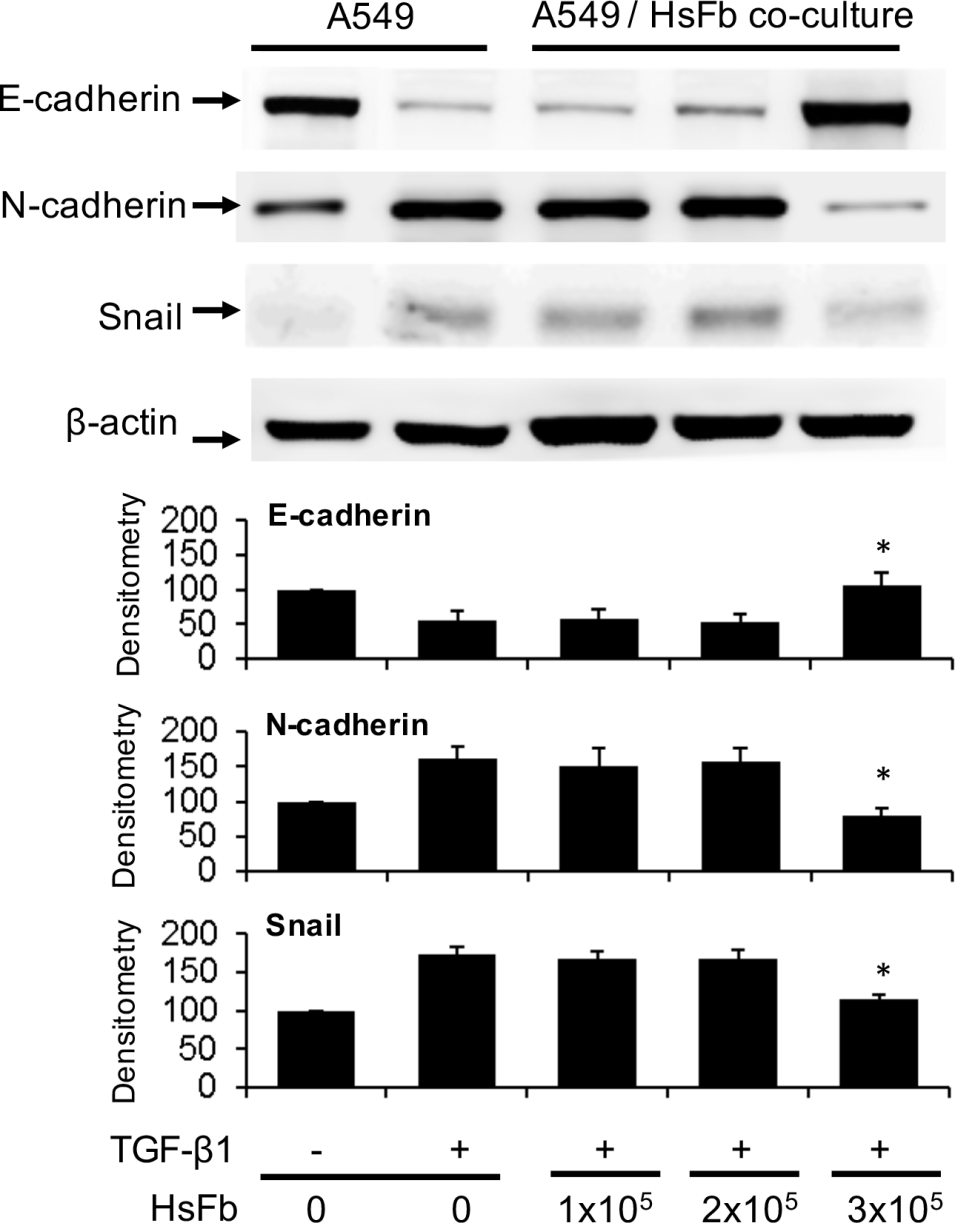
Suppression of A549 EMT by soluble factors of fibroblasts A549 cells (1 × 10^5^ cells) were plated at the lower chamber and Hs68 cells (HsFb) at various cell numbers (1~3 × 10^5^ cells) were plated at the upper chamber. Both chambers were co-incubated with or without TGF-β1 (5 ng/mL) for 48 h. A549 cells were harvested and lysed. E-cadherin, N-cadherin and Snail in the cell lysates were analyzed with Western blotting. The upper panel shows representative blots and the lower panel the densitometry. Error bars denote mean ± SEM (*n*= 3). * indicates *P* < 0.05 compared to control.

To determine whether fibroblasts influence cancer cell EMT, we co-incubated Hs68 fibroblasts with A549 cancer cells in a Boyden chamber. A549 cells (1 × 10^5^ cells) were seeded at the bottom chamber and Hs68 fibroblasts (1~3 × 10^5^ cells) were seeded at the top. After co-incubation of both chambers with or without TGF-β1 (5 ng/mL) for 48 h, A549 cells were harvested and E-cadherin, N-cadherin and Snail in the cell lysates were analyzed by Western blotting. Co-incubation with 3 × 10^5^ fibroblasts prevented E-cadherin decline and N-cadherin increase induced by TGF-β1 which were correlated with change in Snail level (Figure 1). Hs68 fibroblasts at lower cell numbers (1 or 2 × 10^5^ cells) failed to exert a significant effect on Snail, E-cadherin or N-cadherin (Figure 1). These results suggest that fibroblasts release soluble factors to prevent TGF-β1-induced A549 EMT.

### Fibroblasts inhibit A549 EMT via TPH-1-catalyzed 5-MTP production

**Figure 2:**
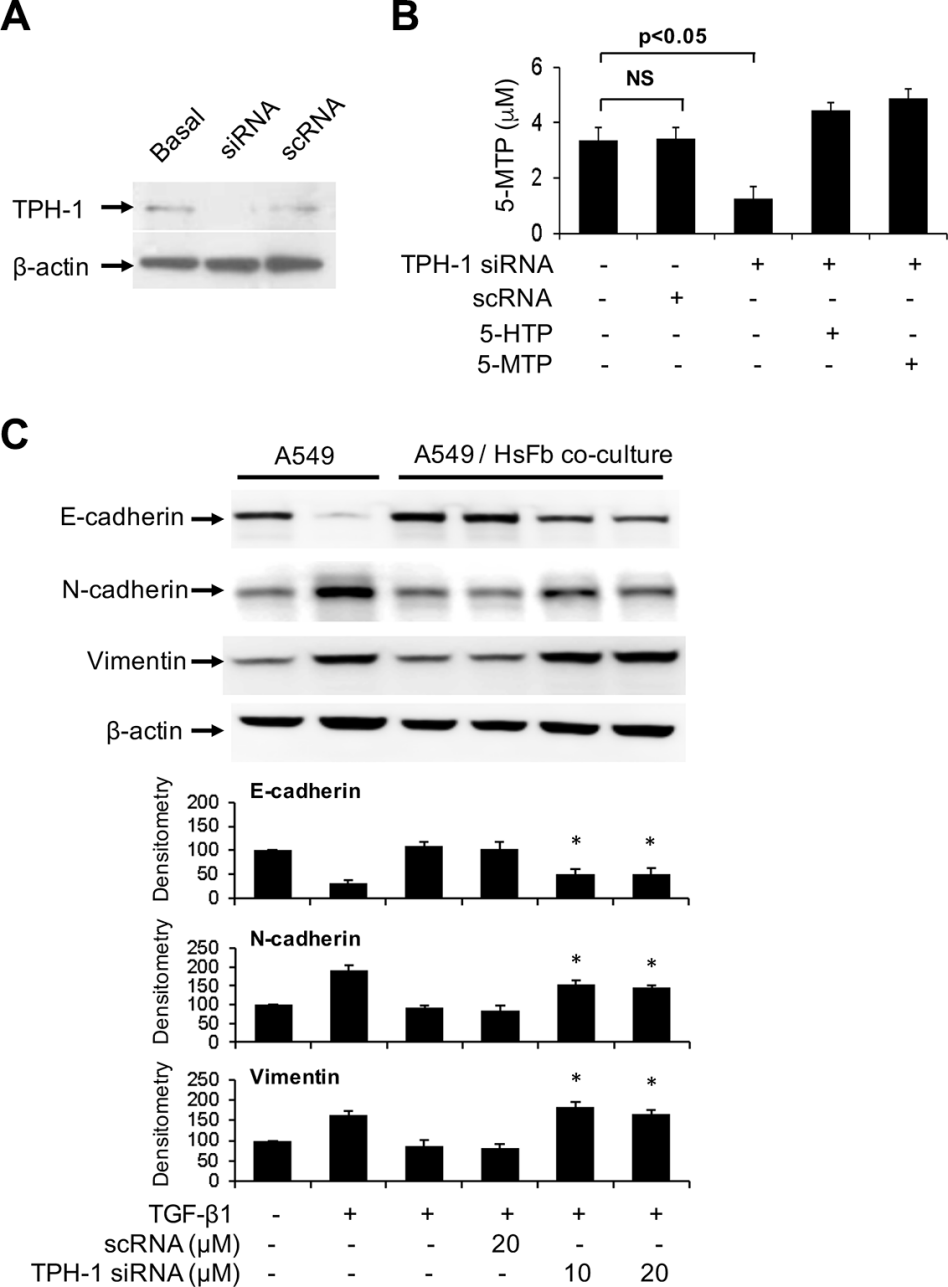
Abrogation of the effect of TPH-1 silenced fibroblasts on A549 EMT (**A**) Hs68 fibroblasts were transfected with TPH-1 siRNA (20 μM) or scRNA (20 μM) for 24 h. TPH-1 proteins in the cell lysates were analyzed by Western blotting. Similar results were observed in two independent experiments. Basal denotes untransfected cells. (**B**) Hs68 fibroblasts were transfected with TPH-1 siRNA or scRNA for 24 h. The conditioned medium was collected and its 5-MTP was measured by ELISA. 5-HTP (5 μM) or 5-MTP (5 μM) was added to TPH-1 silenced fibroblasts and 5-MTP concentration in conditioned medium was measured. Error bars denote mean ± SEM (*n* = 3). * indicates *P* < 0.05 compared to control. NS, not statistically significant. (**C**) HS68 fibroblasts (3 × 10^5^ cells) transfected with TPH-1 siRNA or scRNA were co-incubated with A549 cells (1 × 10^5^ cells) in a Boyden chamber in the presence or absence of TGF-β1 (5 ng/mL) for 48 h. A549 cells were harvested and lysed and EMT markers were analyzed. Upper panel shows a representative Western blot and the lower panel the densitometry. Error bars denote mean ± SEM (*n* = 3). * indicates *P* < 0.05 compared to control.

We reported that fibroblasts release 5-MTP into the conditioned medium which was shown to inhibit A549 COX-2 overexpression, block A549 cell migration and invasion and inhibit A549 cancer growth and lung metastasis in a murine xenograft tumor model [11]. 5-MTP production by fibroblasts is catalyzed by TPH-1. Neither TPH-2 nor aromatic amino acid decarboxylase (also known as dopa decarboxylase or DDC) transcript was detected with RT-PCR analysis (data not shown). Metabolomic analysis of fibroblast conditioned medium by LC-MS-MS failed to detect serotonin, melatonin or their catabolites [11]. These results indicate that Hs68 fibroblasts selectively produce 5-MTP via TPH-1 pathway. To determine whether fibroblasts control A549 EMT via 5-MTP, we transfected Hs68 with TPH-1 siRNA or a control RNA (scRNA) and analyzed A549 EMT in the co-culture system. We confirmed that TPH-1 siRNA reduced TPH-1 protein expression (Figure 2A) and 5-MTP release (Figure 2B). Co-incubation of TPH-1 siRNA-transfected fibroblasts with A549 cells abrogated TGF-β1-induced EMT (Figure 2C). By contrast, scRNA did not change A549 EMT markers. These results indicate that fibroblasts suppress TGF-β1-induced A549 cancer cell EMT through TPH-1 derived 5-MTP.

### 5-MTP inhibits TGF-β1-induced A549 EMT

**Figure 3:**
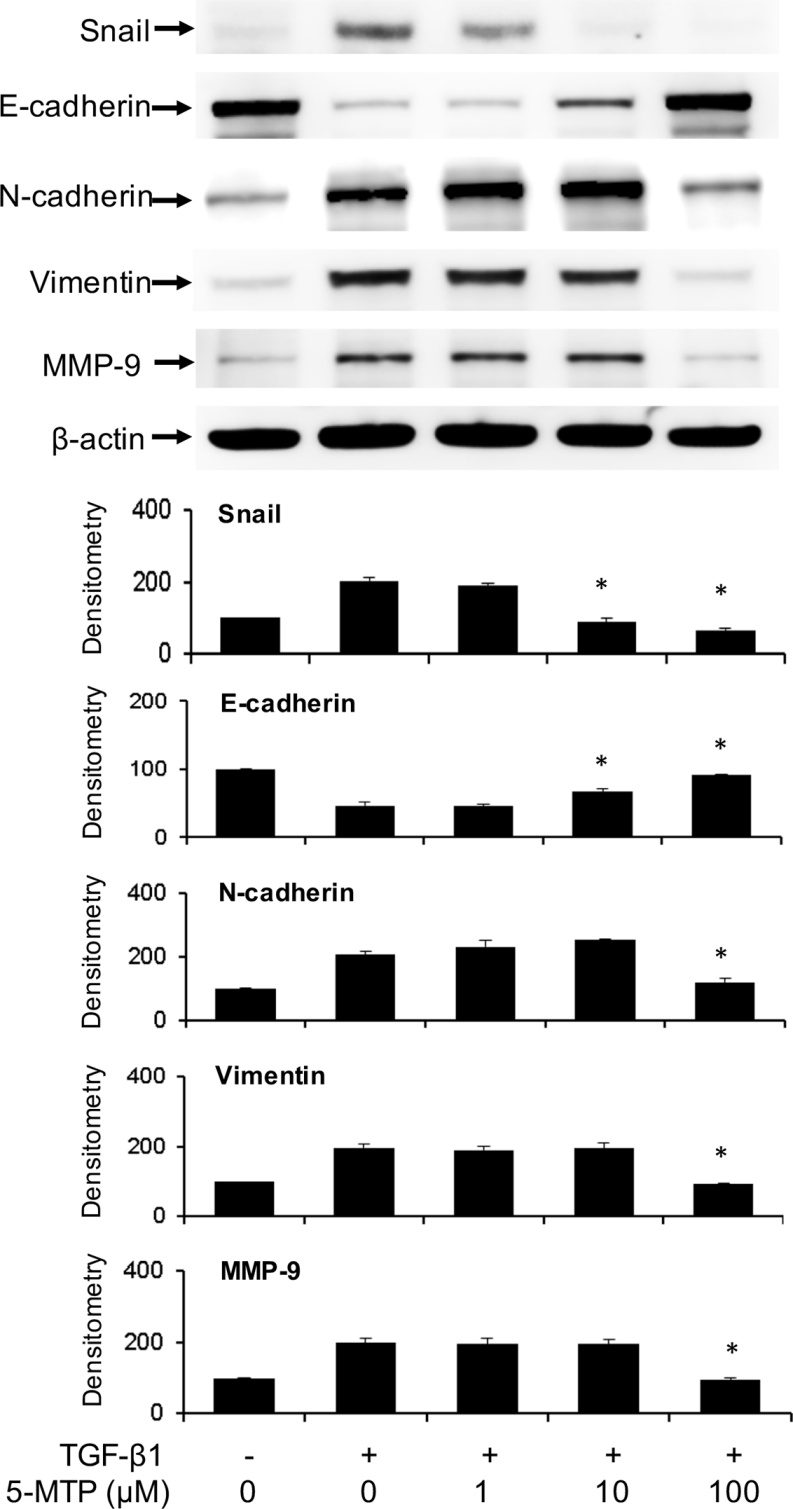
Prevention of TGF-β1-induced A549 EMT by 5-MTP A549 cells were incubated in the presence of 5-MTP or vehicle for 48 h. Cells were lysed and EMT markers were analyzed by Western blotting. The upper panel shows a representative blot and the lower panel the densitometry. Error bars denote mean ± SEM (*n* = 3). * indicates *P* < 0.05 compared to vehicle control.

To ascertain that 5-MTP directly antagonizes TGF-β1-induced A549 EMT, we evaluated the effect of synthetic 5-MTP on Snail, E-cadherin and mesenchymal markers. 5-MTP prevented TGF-β1-induced Snail elevation in a concentration-dependent manner which is closely correlated with rise of E-cadherin (Figure 3). 5-MTP at 10 μM was effective in controlling Snail and maintaining E-cadherin. On the other hand, its control of N-cadherin and vimentin required a higher concentration, i.e. 100 μM (Figure 3). 5-MTP at 10 μM was effective in preventing TGF-β1-induced morphological changes as examined under light microscopy. It preserves A549 epithelial cell appearance with clear cell-cell junction (data not shown).

### 5-MTP administration suppresses cancer cell vimentin expression *in vivo*

**Figure 4:**
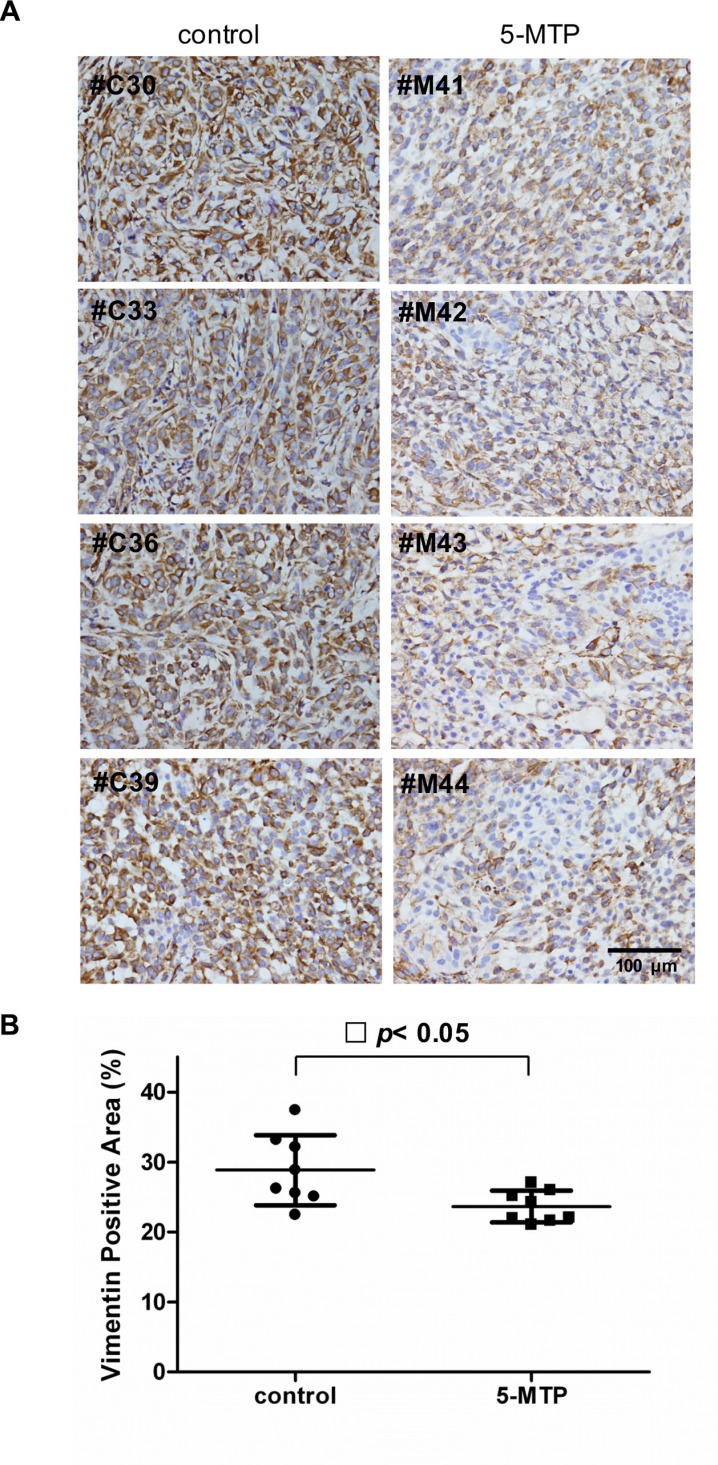
Suppression of cancer cell vimentin by 5-MTP administration *in vivo* (**A**) Images of immunostaining of vimentin of subcutaneous A549 xenograft tumor tissues in 4 control and 4 5-MTP treated mice. (**B**) Quantitative analysis of vimentin-positive areas in tumors of 8 control vs. 8 5-MTP treatment mice. Each dot denotes a mouse lung tissue. Long horizontal lines represent mean values and short lines, ± SD.

We previously reported that intraperitoneal administration of 5-MTP significantly reduced cancer growth and metastasis in a murine xenograft tumor model in which A549 cells were implanted into subcutaneous tissues of SCID mice [11]. In that original study, 10 mice received intraperitoneal infusion of 5-MTP twice weekly and 10 mice received vehicle. Subcutaneous tumor growth was monitored weekly for 7 weeks and on day 50, mice were sacrificed. Lung nodules were inspected and examined under microscope for cancer metastasis to the lung. The results which had been reported previously [11] show that tumor volume at 7 weeks was reduced by ~50% and percentage of lung metastasis was reduced by 40% in the 5-MTP treatment group. In the original study, subcutaneous tumors resected on day 50 were stored at −80°C. Availability of the stored tumor tissues provides an opportunity to evaluate the effect of 5-MTP on cancer EMT *in vivo*. We have thus retrieved the tumors and inspected them for suitability of immunohistochemistry (IHC) analysis. Eight of the 10 control and 8 of 10 5-MTP treatment tumor tissues were feasible for analysis. We chose to analyze tissue level of vimentin because vimentin is suitable for quantitative measurement. Vimentin expression in the cancer tissues was stained by IHC and expression level was determined by measuring vimentin positive area under imaging analysis. When compared with vehicle control, vimentin staining was reduced in the 5-MTP treatment group as illustrated in Figure 4A. Quantitative analysis reveals a significant reduction of vimentin expression in the 5-MTP treatment group (Figure 4B). These results provide evidence for control of A549 cancer cell EMT by 5-MTP *in vivo*.

### 5-MTP inhibits EMT by blocking p38 MAPK activation

**Figure 5:**
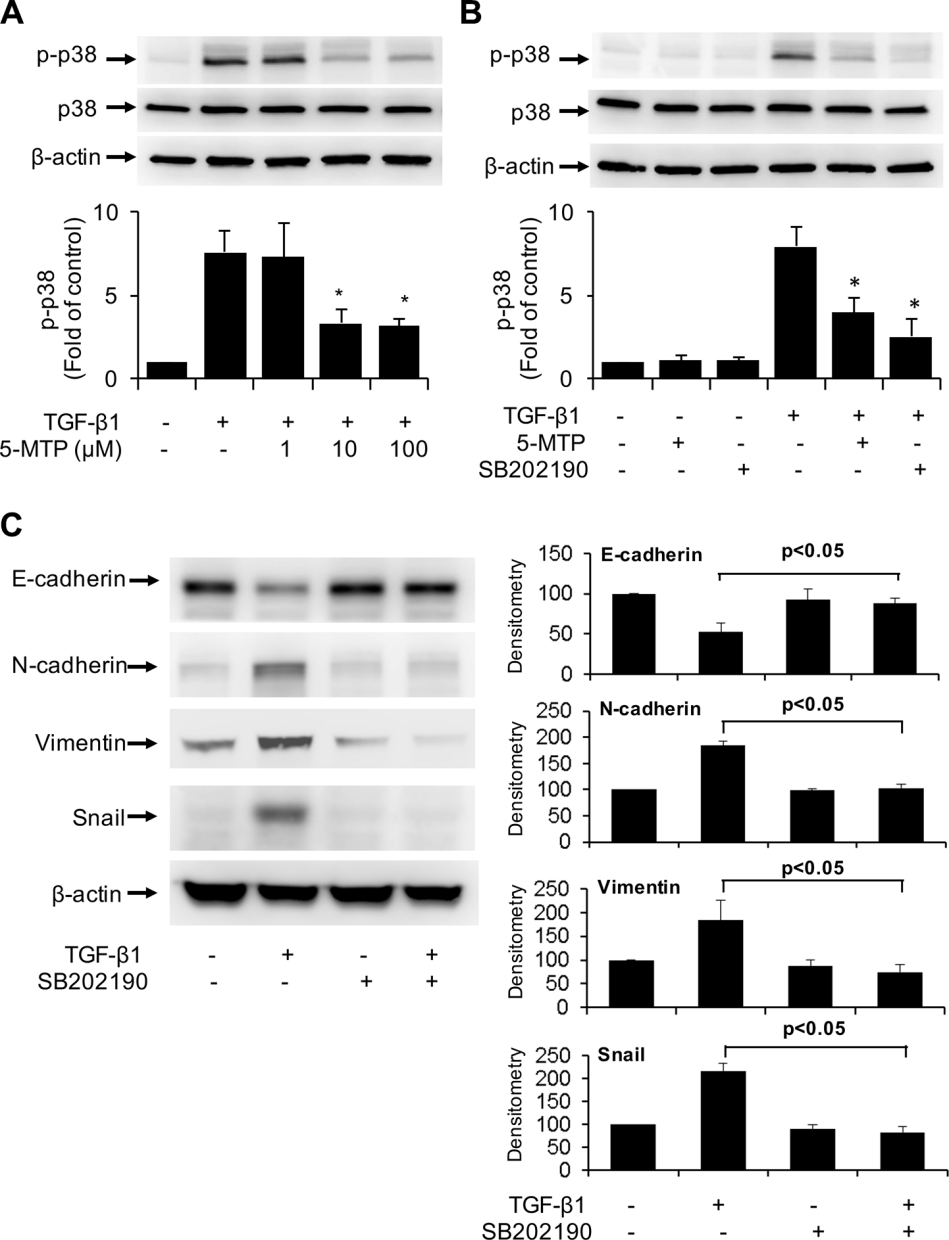
Inactivation of TGF-β1-induced p38 MAPK by 5-MTP (**A**) and (**B**) A549 cells were pretreated with 5-MTP at increasing concentrations (**A**) or 10 μM (**B**) or SB202190 (10 μM) for 30 min followed by TGF-β1 for 15 min. Cells were lysed and p-p38 MAPK (p-p38) and total p38 MAPK (p38) were analyzed by Western blotting. Upper panels show representative blots and lower panels the densitometry of p-p38 blots. Error bars denote mean ± SEM (*n* = 3). * indicates *P* < 0.05 compared to TGF-β1 alone. (**C**) A549 cells were pretreated with SB202190 for 30 min followed by TGF-β1 for 48 h. EMT markers in the cell lysates were analyzed by Western blotting. The left panel shows representative blots and the right panel the densitometry. Error bars denote mean ± SEM (*n* = 3).

TGF-β1-induces EMT via several signaling pathways among which p38 MAPK signaling pathway is considered to be essential [24, 25]. We determined whether the anti-EMT action of 5-MTP may be mediated via blocking p38 MAPK. We confirmed that TGF-β1 induced p38 phosphorylation at 5 min which reached maximal at 10 min and declined thereafter (Figure S1). Pretreatment with 5-MTP resulted in a concentration-dependent reduction of phosphorylated p38 MAPK (p-p38) with maximal inhibition at 10 μM (Figure 5A). For comparison, we evaluated the effect of SB202190, an inhibitor of p38, on p-p38. TGF-β1-induced p-p38 was abrogated by SB202190 (Figure 5B). These results suggest that 5-MTP blocks TGF-β1-induced p38 MAPK activation to an extent comparable to selective chemical inhibitor of p38. We next determined whether TGF-β1-induced EMT is affected by SB202190. TGF-β1-induced upregulation of Snail and downregulation of E-cadherin was abrogated by SB202190 (Figure 5C). TGF-β1-induced increase in mesenchymal markers such as N-cadherin and vimentin was blocked by the p38 MAPK inhibitor (Figure 5C). These results suggest that 5-MTP inhibits TGF-β1-induced EMT by suppressing p38 MAPK activation.

### 5-MTP blocks TGF-β1-induced NF-κB activation

**Figure 6:**
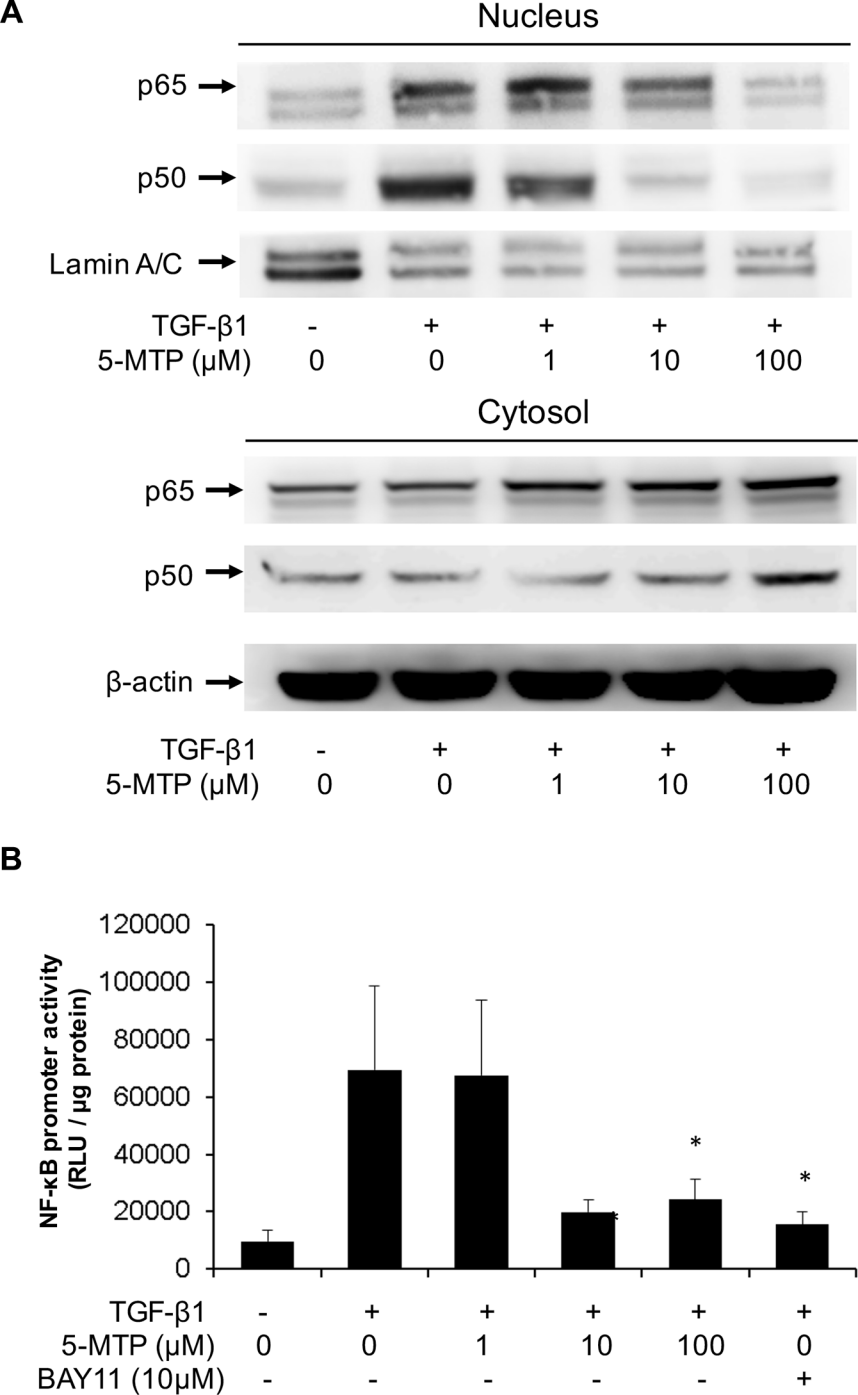
5-MTP inhibits TGF-β1-induced NF-κB activation in A549 cells (**A**) A549 cells were treated with 5-MTP for 30 min followed by TGF-β1 for 6 h. Cytosolic and nuclear fractions were prepared. p65 and p50 NF-κB in both fractions were analyzed with Western blotting. The Western blots are representative of two independent experiments with similar results. (**B**) A549 cells transfected with NF-κB promoter-luciferase reporter construct were treated with 5-MTP followed by TGF-β1 for 6 h. Promoter activity was analyzed as luciferase activity expressed as relative light unit (RLU). Bay11, a NF-κB inhibitor was included as positive control. Error bars denote mean ± SD (*n* = 3). * indicates *P < 0.05.*

It was reported that NF-κB activation is essential for breast cancer cell EMT induced by TGF-β1 [26]. NF-κB activation was reported to promote EMT of pancreatic cancer cells [27]. NF-κB mediates EMT of cancer cells by increasing expression of Snail or Twist 1 [28]. We determined whether 5-MTP affects TGF-β1-induced NF-κB activation. 5-MTP blocked TGF-β1-induced p65 and p50 nuclear translocation in a concentration-dependent manner. 5-MTP at 10 μM reduced p50 translocation to a greater extent than p65 translocation and at 100 μM it completely blocked p50 and p65 nuclear translocation (Figure 6A). TGF-β1-induced NF-κB promoter activity was reduced by 5-MTP in a concentration-dependent manner comparable to reduction of NF-κB nuclear translocation. Bay 11, an inhibitor of NF-κB transactivation, was included as a positive control which reduced TGF-β1-induced NF-κB promoter activity almost to the basal level (Figure 6B). These results suggest that 5-MTP blocks TGF-β1-induced A549 EMT by inhibiting NF-κB transactivation and NF-κB mediated Snail expression. As pro-inflammatory mediator and mitogenic factor-induced COX-2 expression depends on NF-κB, we next determined whether COX-2 regulates EMT.

### COX-2 is not required for TGF-β1-induced EMT in A549 cells

COX-2 overexpression has been implicated in regulation of EMT of several types of cancer cells [29, 30]. Selective COX-2 inhibitors were reported to reverse EMT [31]. By contrast, celecoxib was reported to enhance EMT in human lung cancer cells [32]. Since 5-MTP was discovered as a COX-2 suppressing factor, we determined whether its EMT inhibitory action is mediated via COX-2. Contrary to stimulation of COX-2 expression in breast and colorectal cancer cells, TGF-β1 reduced COX-2 expression in A549 cells (Figure S2). Inhibition of COX-2 with a selective inhibitor NS398 exerted little effect on A549 E-cadherin, N-cadherin or vimentin (Figure S3). These results indicate that COX-2 does not play an important role in TGF-β1-induced EM transition in A549 cells.

### Inhibition of p300 HAT does not alter TGF-β1-induced EMT in A549 cells

**Figure 7:**
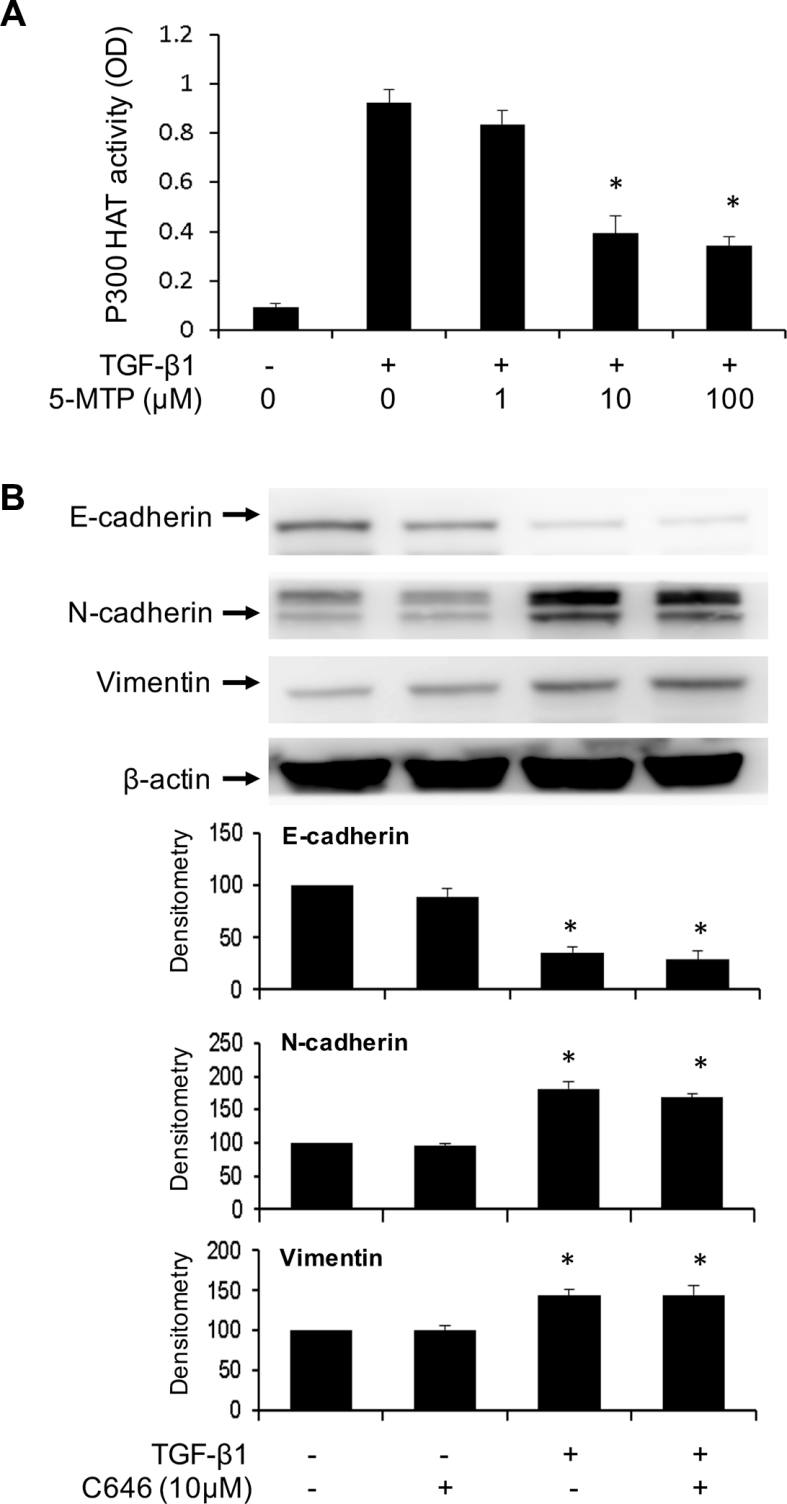
p300 HAT is inhibited by 5-MTP but its inhibition does not influence EMT (**A**) A549 cells were treated with 5-MTP for 30 min followed by TGF-β1 for 24 h. p300 proteins were isolated by immunoprecipitation and HAT activity of IP-isolated p300 was measured. Error bars indicate mean ± SEM (*n* = 3). (**B**) A549 cells were pretreated with C646 for 30 min followed by TGF-β1 for 48 h. EMT markers were analyzed by Western blotting. Upper panels show representative Western blots and lower panels show densitometry. Error bars indicate mean ± SEM (*n* = 3). * indicates *P* < 0.05.

p300 transcriptional co-activator possesses HAT activity which acetylates histone to open up chromatin structure and acetylates myriad transcriptional activators such as NF-κB and Smad to enhance their transactivation function [33]. Acetylation of Smad 2/3 was reported to enhance Smad-mediated Snail transcription [34, 35]. p300 HAT is well known to acetylate NF-κB and increase NF-κB mediated gene expression including COX-2 and Snail [36]. NF-κB and Smad are the key transcriptional pathways to drive EMT via Snail and Twist upregulation. Soluble factors in proliferating fibroblasts conditioned medium and 5-MTP were reported to inhibit p300 HAT activation induced by phorbol esters [12]. We determined here whether TGF-β1 induces p300 HAT activation and whether 5-MTP influences the activation. p300 proteins were isolated from A549 lysates by immunoprecipitation and HAT activity of the purified proteins was analyzed. TGF-β1 increased p300 HAT activity by >10 folds over the basal control and pretreatment with 5-MTP significantly reduced TGF-β1-induced p300 HAT activity (Figure 7A). We next evaluated the effect of C646, a chemical inhibitor of p300 HAT on TGF-β1-induced EMT. C646 had no significant effect on TGF-β1-induced changes in E-cadherin, N-cadherin or vimentin (Figure 7B). These results indicate that although TGF-β1 increases p300 HAT activity, its induction of EMT is independent of p300 HAT. Furthermore, despite its inhibitory action on p300 HAT, 5-MTP blocks EMT by mechanisms independent of p300 HAT.

## DISCUSSION

Our findings indicate that human foreskin fibroblasts release soluble factors to reduce A549 cancer cell EMT. Since silencing of TPH-1, an enzyme required for 5-MTP production in fibroblasts, loses the control of TGF-β1-induced EMT and addition of synthetic 5-MTP restores the control, 5-MTP is a key component of the soluble factors. In this study, we chose A549 cells as a cell model to prove the concept that fibroblast-derived 5-MTP inhibits cancer cell EMT induced by TGF-β1 because TGF-β1-induced EMT in A549 cells has been extensively characterized [37, 38]. However, further studies are needed to evaluate the effect of 5-MTP on inhibiting EMT in other cancer cells and on suppressing EMT in cancer cells induced by other growth factors. The *in vivo* relevance is supported by the demonstration in the xenograft murine model that administration of 5-MTP reduces A549 cancer cell vimentin expression. These results show for the first time the identification of a novel tryptophan metabolite, i.e. 5-MTP as a soluble factor released by normal fibroblasts to control cancer cell EMT.

We have previously reported that 5-MTP synthesis in fibroblasts depends on TPH-1. TPH-1 catalyzes the formation of 5-hydroxytryptophan (5-HTP), which is a common precursor for synthesis of not only 5-MTP but also serotonin, melatonin and their catabolites. Conversion of 5-HTP to serotonin and melatonin requires a decarboxylase step whereas conversion of 5-HTP to 5-MTP does not. Since fibroblasts do not have detectable AAA (aromatic amino acid) decarboxylase, it is unlikely that fibroblasts are capable of producing serotonin or melatonin. This was supported by our previous metabolomic analysis of fibroblast conditioned medium. Taken together, these results indicate that fibroblasts use TPH-1 to produce 5-MTP and loss of EMT control in TPH-1 silenced fibroblasts is attributed to deficient 5-MTP production.

TGF-β1 induces EMT by engaging with its specific receptors which transmit the message via a number of signaling pathways [24, 25]. One of the major pathways is p38 mitogen-activated protein kinase (MAPK). It was reported that TGF-β1-activated p38 MAPK is required for cancer cell mesenchymal transdifferentiation and cell migration [24, 25] and inhibition of p38 MAPK reverses EMT [39]. We determined whether p38 MAPK activation is required for TGF-β1-induced EMT in A549 cells and whether 5-MTP blocks p38 MAPK activation. Consistent with the reported results in other cell types, TGF-β1 activates p38 MAPK maximally at 10 min. Inhibition of TGF-β1-induced p38 MAPK activation prevents Snail overexpression accompanied by preservation of E-cadherin. 5-MTP inhibits TGF-β1-induced p38 MAPK at concentrations comparable to its inhibition of Snail expression and maintaining E-cadherin level (Figure 5A vs. Figure 3). These results suggest that 5-MTP controls TGF-β1-induced EMT by blocking p38 MAPK signaling. It is unclear how 5-MTP blocks p38 MAPK. One possibility is through the induction of phosphatases that antagonize p38 MAPK such as MAP kinase phosphatase-1 (MKP-1) [40]. Further studies are needed to elucidate the mechanism by which 5-MTP controls Snail expression.

TGF-β1 induces cancer cell EMT via several transcriptional mechanisms [13], among which NF-κB transactivation was reported to be essential for mesenchymal switch of breast cancer cells [26, 41] and involved in EMT induction in other cancer cell types [42]. NF-κB transactivation results in expression of EMT-inducing genes notably Snail which is a key transcription factor mediating E-cadherin suppression and induction of mesenchymal genes such as N-cadherin, vimentin and MMP [42–44]. In this study, we confirmed that TGF-β1 activates NF-κB in A549 cells and provide novel information regarding the inhibition of TGF-β1-induced NF-κB transactivation by 5-MTP. 5-MTP at 10 μM is effective in reducing NF-κB promoter activity to almost the basal level which is correlated with blocking of p50 translocation from cytosol to nucleus. We have previously reported that 5-MTP blocks binding of NF-κB to COX-2 promoter in fibroblasts. Taken together, these results suggest that 5-MTP inhibits A549 EMT by blocking NF-κB nuclear translocation and transactivation. NF-κB was reported to mediate TGF-β1-induced vimentin which was identified as an independent predictor of prostate cancer recurrence [44]. Our findings that 5-MTP administration suppresses vimentin expression in A549 xenograft cancer cells are consistent with the notion that 5-MTP exerts its anti-cancer actions by inhibiting NF-κB activation. There is cross-talk between NF-κB activation and p38 MAPK pathway. The p38 MAPK pathway was reported to positively regulate NF-κB transactivation through activation of mitogen- and stress-activated protein kinase-1 (MSK-1) [45]. Furthermore, p38 MAPK phosphorylates histone H3 at the NF-κB binding motif region of the promoter of pro-inflammatory cytokines resulting in chromatin modification and increasing accessibility of NF-κB binding sites [46]. Thus, p38 MAPK activation enhances NF-κB transactivation by multiple mechanisms and the inhibitory action of 5-MTP on NF-κB transactivation may be medicated by blocking p38 MAPK activation. Alternatively, 5-MTP may inhibit p38 and NF-κB through upregulating Wip-1 phosphatase. Wip-1 dephosphorylates p65 subunit thereby reducing NF-κB transactivation [47]. In addition, Wip-1 dephosphorylates p38 and therefore interferes with p38 MAPK activation [47]. Thus, in addition to MKP-1, Wip-1 could be involved in 5-MTP mediated inhibition of p38 MAPK and NF-κB activation.

p300 and its paralog CREB bending protein (CBP) were reported to enhance TGF-β1 signaling [34] and modulate cancer cell EMT [48, 49]. Expression of high p300 levels was reported to predict shortened survival in patients with hepatocellular carcinoma [49]. We have observed that 5-MTP inhibits p300 HAT in quiescent fibroblasts [12]. Hence, we were curious whether 5-MTP exerts its counter-EMT effect in A549. Similar to its effect in fibroblasts, 5-MTP inhibits TGF-β1-induced p300 HAT in A549 cells in a concentration-dependent manner. However, inhibition of p300 HAT activity with an inhibitor, C646, did not influence A549 EMT, suggesting that p300 HAT is not required for TGF-β1-induced EMT. In separate experiments, silencing of p300 with siRNA also did not alter TGF-β1-induced EMT (data not shown). The role of p300 in EMT remains unresolved. Silencing of p300 was reported to induce EMT in HCT 116 cells [48].

COX-2 is overexpressed in many types of human cancers including lung cancer [50–52]. COX-2 overexpression promotes cancer growth and enhances cancer invasion and metastasis [53, 54] and has been implicated in cancer cell EMT [29, 30]. However, our results show that TGF-β1-induced EMT in A549 cells is independent of COX-2. In fact, TGF-β1 reduces COX-2 expression in A549 cells. Takai et al. reported similar results about COX-2 suppression by TGF-β1 in A549 cells [55]. These data imply that 5-MTP controls A549 EMT in a COX-2 independent manner.

In summary, we provide evidence for suppressing lung cancer cell EMT by fibroblasts through the production of a novel tryptophan metabolite, i.e. 5-MTP. Our findings indicate that 5-MTP controls EMT via inhibition of p38 MAPK which is required for TGF-β1-induced A549 EMT. p38 MAPK is known to activate NF-κB which is essential for EMT. We conclude that 5-MTP is an innate metabolite to control cancer cell mesenchymal transdifferentiation, migration and metastasis.

## MATERIALS AND METHODS

### Materials

L-5-MTP (5-MTP) was custom-synthesized by Asta Tech Inc. Its purity was verified by LC-MS. Rabbit monoclonal antibodies against E-cadherin, Snail, vimentin, phospho-p38 (Thr180/Tyr182) and p38 were purchased from Cell Signaling Technology. Rabbit monoclonal antibodies against N-cadherin and MMP 9 were purchased from Abcam. Rabbit monoclonal antibodies against p65 and p50 were purchased from Santa Cruz. Rabbit polyclonal antibodies against 5-MTP was purchased from Abcam. Horseradish peroxidase-conjugated anti-mouse and anti-rabbit IgG were purchased from Santa Cruz. TGF-β1 was purchased from PeproTech. SB202190 was purchased from Cell Signaling Technology. NS398 and Bay11 were purchased from Sigma-Aldrich. p300 HAT inhibitor, C646, was purchased from EMD Millipore.

### Cell culture and treatment

Human Hs68 foreskin fibroblasts and human A549 lung adenocarcinoma cells were obtained from American Type Culture Collection and cultured in Dulbecco's modified Eagle's medium supplemented with 10% FBS at 37°C in a 5% CO_2_ incubator. Hs68 cells up to 15 passages were used for all experiments. For EMT experiments, 80–90% confluent cells were washed with PBS and cultured in serum-free medium for 24 h and then incubated with 5 ng/mL TGF-β1 for 48 h in the absence or presence of 5-MTP. For co-culture experiments, Hs68 fibroblasts were seeded on the upper chamber and A549 cells were seeded at the lower chamber of a Boyden chamber with 8-μm pores (Corning). The cells were co-incubated with or without 5 ng/mL TGF-β1 for 48 h. A549 cells were then harvested and EMT proteins were analyzed by Western blotting.

### NF-κB transactivation assay

NF-κB transactivation activity was analyzed using pGL 4.32 vector from Promega. The pGL 4.32 (luc2P/NF-κB-RE/Hygro) vector contains 5 copies of an NF-κB response element which drives transcription of the luciferase reporter gene luc2P. Transfection of the NF-κB promoter-luciferase reporter construct into the Hs68 fibroblasts was performed as previously described [12]. In brief, 4 μg of the promoter vector was mixed with 10 μL of lipofectamine 2000 (Invitrogen). The mixture was slowly added to cells in a 6-well plate and incubated for 24 h. After treatment, cells were lysed and luciferase activity was measured using an assay kit from Promega with a luminometer (TD-20/20, Promega).

### siRNA experiments

The target siRNA sequences used to silence human TPH-1 comprise three different siRNA duplexes (Santa Cruz): 5′-CUG UGA AUC UAC CAG AUA ATT-3′; 5′-CCA ACA GAG UUC UGA UGU ATT-3′ and 5′-GGA AUG UCU UAU CAC AAC UTT-3′. The negative control (scRNA) sequence was 5′-UUC UCC GAA CGU GUC ACG UTT-3′. Cells were transfected with siRNA duplexes using Lipofectamine 2000 (Invitrogen) as previously described [12]. In brief, siRNA or scRNA plasmids (1 μg DNA in 10 μL) and transfection reagent (5 μL) were incubated in 200 μL serum-free medium for 20 min at room temperature. The mixture was added dropwise to each well and incubated for 7 h. The cells were then washed twice with PBS and maintained in culture medium for 24 h.

### Analysis of p300 HAT activity

Nuclear extracts were prepared from Hs68 fibroblasts and p300 proteins were isolated by immunoprecipitation with anti-p300 antibodies (Santa Cruz). Isolated p300 proteins were incubated with a reaction mixture containing 10 μL assay buffer and 10 μL acetyl-CoA at a final concentration of 100 μM at 30°C for 1 h. Tetramethylbenzidine substrate mixture was added and substrate acetylation was analyzed at 450 nm and 570 nm. Optical density values at 570 nm were subtracted to remove any well to well plate variation. Histone H4 acetylation was included as positive controls.

### Measurement of 5-MTP with enzyme-immunoassay

The ELISA assay was modified from that previously described [11]. In brief, a mixture of 5-MTP standards or samples and 5-MTP conjugated with horseradish peroxidase were added to 96-well microtiter plates precoated with 5-MTP antibodies and incubated at 4°C overnight. After washing, tetramethylbenzidine was added and incubated at room temperature for 30 min. Reaction was stopped with 0.1 mM H_2_SO_4_ and the product was analyzed at 450 nm. The calibration curve for each experiment was constructed by using pure 5-MTP at concentrations from 0.1 to 50 μM.

### Reverse transcription-polymerase chain reaction (RT-PCR)

Cells were lysed with 1 mL Trizol/well for 5 min and RNA was purified from the lysates with Direct-zol RNA MiniPrep kit (Zymo research). First-strand cDNA was synthesized by incubating 1 μg RNA and SuperScript III First-Strand Synthesis SuperMix (Invitrogen) for 30 min at 50°C. TPH-1, TPH-2, or DDC DNA was amplified by mixing 1 μL cDNA, 1 μL primers (10 μM each) and 47 μL HotStar PCR SuperMix (GeneDirex) for 35 cycles (94°C for 20 sec, 57°C for 20 sec, 72°C for 45 sec) with a MultiGene Thermal Cycler system (Labnet International). The primer sequences for TPH-1 are: forward, CGT CCT GTG GCT GGT TAC TTA and reverse, AGT AGC ACG TTG CCA GTT TTT G. The primer sequences for TPH- 2 are: forward, TTG GAG AAT TAA AGC ACG CCC and reverse, ACA ATG AGT GGT TAT CTG CCA T. The primer sequences for DDC are: forward, GCC ATC AGG ATT CAG GGC TT and reverse, GCC CCA GAA TGA CTT CCA CA.

### Immunohistochemistry

The mouse tumor tissue in paraffin block was sectioned and immunostained with vimentin antibody (Abcam) by IHC Select Immunoperoxidase Secondary Detection System (Millipore). The images were photographed in 200 × magnification. The DAB image was first separated from hematoxylin image using color deconvolution plugin in Image J (NIH), and the vimentin-positive area was quantified by ImageJ.

### Immunoblotting

Cells were lysed in RIPA lysis buffer (Upstate) containing protease inhibitor cocktail (Roche) and centrifuged at 16,000 × g for 10 min. Protein concentration was determined by protein assay (Bio-Rad). Lysates containing 20 μg proteins were denatured at 95°C for 10 min before being subjected to 4–15% SDS-PAGE and then transferred to polyvinylidene difluoride membranes (Millipore). For detection, membranes were blocked with 5% bovine serum albumin in TBS containing 0.05% Tween 20 for 1 h at 22°C and then incubated with 1:1000 diluted primary antibodies overnight at 4°C. Membranes were incubated with 1:3000 diluted horseradish peroxidase-linked secondary antibodies for 1 h at 22°C and then developed with enhanced chemiluminescence substrate (Thermo Scientific). The blot was exposed with a luminescent image analyzer (ImageQuant LAS 4000, GE Healthcare) and quantified by ImageJ software (National Institute of Health).

### Cytoplasmic and nuclear fractionation

Nuclear–cytoplasmic fractionation was conducted using the NE-PER Nuclear and Cytoplasmic Extraction Reagents kit (Thermo Scientific) according to the manufacturer's protocol. In brief, cells were harvested and centrifuged at 1,500 × g for 5 min. The pellet was resuspended in CER I solution for 10 min and then mixed with CER II solution for 1 min. After centrifugation at 16,000 × g for 5 min, the supernatant (cytoplasmic fraction) was collected and the pellet was washed and then resuspended in NER solution. After centrifugation at 16,000 × g for 10 min, the supernatant (nuclear fraction) was collected. The extracted fractions were stored at −80°C until use.

### Statistical analysis

Values were expressed as mean ± standard error (SEM) or standard deviation (SD) as indicated. Differences between groups were analyzed using One Way ANOVA with SigmaStat software (Systat Software, Inc.). *P < 0.05* was considered statistically significant.

## SUPPLEMENTARY MATERIALS FIGURES



## References

[1] Martin P (1997). Wound healing—aiming for perfect skin regeneration. Science.

[2] Sartore S, Chiavegato A, Faggin E, Franch R, Puato M, Ausoni S, Pauletto P (2001). Contribution of adventitial fibroblasts to neointima formation and vascular remodeling: from innocent bystander to active participant. Circ Res.

[3] Kalluri R, Zeisberg M (2006). Fibroblasts in cancer. Nat Rev Cancer.

[4] Orimo A, Weinberg RA (2006). Stromal fibroblasts in cancer: a novel tumor-promoting cell type. Cell Cycle.

[5] Sugimoto H, Mundel TM, Kieran MW, Kalluri R (2006). Identification of fibroblast heterogeneity in the tumor microenvironment. Cancer Biol Ther.

[6] Augsten M (2014). Cancer-associated fibroblasts as another polarized cell type of the tumor microenvironment. Front Oncol.

[7] Ozdemir BC, Pentcheva-Hoang T, Carstens JL, Zheng X, Wu CC, Simpson TR, Laklai H, Sugimoto H, Kahlert C, Novitskiy SV, De Jesus-Acosta A, Sharma P, Heidari P (2014). Depletion of carcinoma-associated fibroblasts and fibrosis induces immunosuppression and accelerates pancreas cancer with reduced survival. Cancer Cell.

[8] Stoker MG, Shearer M, O'Neill C (1966). Growth inhibition of polyoma-transformed cells by contact with static normal fibroblasts. J Cell Sci.

[9] Paland N, Kamer I, Kogan-Sakin I, Madar S, Goldfinger N, Rotter V (2009). Differential influence of normal and cancer-associated fibroblasts on the growth of human epithelial cells in an *in vitro* cocultivation model of prostate cancer. Mol Cancer Res.

[10] Flaberg E, Markasz L, Petranyi G, Stuber G, Dicso F, Alchihabi N, Olah E, Csizy I, Jozsa T, Andren O, Johansson JE, Andersson SO, Klein G (2011). High-throughput live-cell imaging reveals differential inhibition of tumor cell proliferation by human fibroblasts. Int J Cancer.

[11] Cheng HH, Kuo CC, Yan JL, Chen HL, Lin WC, Wang KH, Tsai KK, Guven H, Flaberg E, Szekely L, Klein G, Wu KK (2012). Control of cyclooxygenase-2 expression and tumorigenesis by endogenous 5-methoxytryptophan. Proc Natl Acad Sci U S A.

[12] Cheng HH, Wang KH, Chu LY, Chang TC, Kuo CC, Wu KK (2014). Quiescent and proliferative fibroblasts exhibit differential p300 HAT activation through control of 5-methoxytryptophan production. PLoS One.

[13] Lamouille S, Xu J, Derynck R (2014). Molecular mechanisms of epithelial-mesenchymal transition. Nat Rev Mol Cell Biol.

[14] Kalluri R, Weinberg RA (2009). The basics of epithelial-mesenchymal transition. J Clin Invest.

[15] Tsai JH, Yang J (2013). Epithelial-mesenchymal plasticity in carcinoma metastasis. Genes Dev.

[16] Zavadil J, Bottinger EP (2005). TGF-beta and epithelial-to-mesenchymal transitions. Oncogene.

[17] Batlle E, Sancho E, Franci C, Dominguez D, Monfar M, Baulida J, Garcia De Herreros A (2000). The transcription factor snail is a repressor of E-cadherin gene expression in epithelial tumour cells. Nat Cell Biol.

[18] Comijn J, Berx G, Vermassen P, Verschueren K, van Grunsven L, Bruyneel E, Mareel M, Huylebroeck D, van Roy F (2001). The two-handed E box binding zinc finger protein SIP1 downregulates E-cadherin and induces invasion. Mol Cell.

[19] Yang J, Mani SA, Donaher JL, Ramaswamy S, Itzykson RA, Come C, Savagner P, Gitelman I, Richardson A, Weinberg RA (2004). Twist, a master regulator of morphogenesis, plays an essential role in tumor metastasis. Cell.

[20] Kim ES, Kim MS, Moon A (2004). TGF-beta-induced upregulation of MMP-2 and MMP-9 depends on p38 MAPK, but not ERK signaling in MCF10A human breast epithelial cells. Int J Oncol.

[21] Vered M, Dayan D, Yahalom R, Dobriyan A, Barshack I, Bello IO, Kantola S, Salo T (2010). Cancer-associated fibroblasts and epithelial-mesenchymal transition in metastatic oral tongue squamous cell carcinoma. Int J Cancer.

[22] Zhuang J, Lu Q, Shen B, Huang X, Shen L, Zheng X, Huang R, Yan J, Guo H (2015). TGFbeta1 secreted by cancer-associated fibroblasts induces epithelial-mesenchymal transition of bladder cancer cells through lncRNA-ZEB2NAT. Sci Rep.

[23] Rasanen K, Vaheri A (2010). Activation of fibroblasts in cancer stroma. Exp Cell Res.

[24] Bakin AV, Rinehart C, Tomlinson AK, Arteaga CL (2002). p38 mitogen-activated protein kinase is required for TGFbeta-mediated fibroblastic transdifferentiation and cell migration. J Cell Sci.

[25] Gui T, Sun Y, Shimokado A, Muragaki Y (2012). The Roles of Mitogen-Activated Protein Kinase Pathways in TGF-beta-Induced Epithelial-Mesenchymal Transition. J Signal Transduct.

[26] Huber MA, Azoitei N, Baumann B, Grunert S, Sommer A, Pehamberger H, Kraut N, Beug H, Wirth T (2004). NF-kappaB is essential for epithelial-mesenchymal transition and metastasis in a model of breast cancer progression. J Clin Invest.

[27] Maier HJ, Schmidt-Strassburger U, Huber MA, Wiedemann EM, Beug H, Wirth T (2010). NF-kappaB promotes epithelial-mesenchymal transition, migration and invasion of pancreatic carcinoma cells. Cancer Lett.

[28] Li CW, Xia W, Huo L, Lim SO, Wu Y, Hsu JL, Chao CH, Yamaguchi H, Yang NK, Ding Q, Wang Y, Lai YJ, LaBaff AM (2012). Epithelial-mesenchymal transition induced by TNF-alpha requires NF-kappaB-mediated transcriptional upregulation of Twist1. Cancer Res.

[29] Neil JR, Johnson KM, Nemenoff RA, Schiemann WP (2008). Cox-2 inactivates Smad signaling and enhances EMT stimulated by TGF-beta through a PGE2-dependent mechanisms. Carcinogenesis.

[30] Bocca C, Ievolella M, Autelli R, Motta M, Mosso L, Torchio B, Bozzo F, Cannito S, Paternostro C, Colombatto S, Parola M, Miglietta A (2014). Expression of Cox-2 in human breast cancer cells as a critical determinant of epithelial-to-mesenchymal transition and invasiveness. Expert Opin Ther Targets.

[31] Adhim Z, Matsuoka T, Bito T, Shigemura K, Lee KM, Kawabata M, Fujisawa M, Nibu K, Shirakawa T (2011). *In vitro* and *in vivo* inhibitory effect of three Cox-2 inhibitors and epithelial-to-mesenchymal transition in human bladder cancer cell lines. Br J Cancer.

[32] Wang ZL, Fan ZQ, Jiang HD, Qu JM (2013). Selective Cox-2 inhibitor celecoxib induces epithelial-mesenchymal transition in human lung cancer cells via activating MEK-ERK signaling. Carcinogenesis.

[33] Goodman RH, Smolik S (2000). CBP/p300 in cell growth, transformation, and development. Genes Dev.

[34] Janknecht R, Wells NJ, Hunter T (1998). TGF-beta-stimulated cooperation of smad proteins with the coactivators CBP/p300. Genes Dev.

[35] Ko H, So Y, Jeon H, Jeong MH, Choi HK, Ryu SH, Lee SW, Yoon HG, Choi KC (2013). TGF-beta1-induced epithelial-mesenchymal transition and acetylation of Smad2 and Smad3 are negatively regulated by EGCG in human A549 lung cancer cells. Cancer Lett.

[36] Wu Y, Zhou BP (2010). TNF-alpha/NF-kappaB/Snail pathway in cancer cell migration and invasion. Br J Cancer.

[37] Kim J, Hwan Kim S (2013). CK2 inhibitor CX-4945 blocks TGF-beta1-induced epithelial-to-mesenchymal transition in A549 human lung adenocarcinoma cells. PLoS One.

[38] Kin R, Kato S, Kaneto N, Sakurai H, Hayakawa Y, Li F, Tanaka K, Saiki I, Yokoyama S (2013). Procyanidin C1 from Cinnamomi Cortex inhibits TGF-beta-induced epithelial-to-mesenchymal transition in the A549 lung cancer cell line. Int J Oncol.

[39] Antoon JW, Nitzchke AM, Martin EC, Rhodes LV, Nam S, Wadsworth S, Salvo VA, Elliott S, Collins-Burow B, Nephew KP, Burow ME (2013). Inhibition of p38 mitogen-activated protein kinase alters microRNA expression and reverses epithelial-to-mesenchymal transition. Int J Oncol.

[40] Kiemer AK, Weber NC, Furst R, Bildner N, Kulhanek-Heinze S, Vollmar AM (2002). Inhibition of p38 MAPK activation via induction of MKP-1: atrial natriuretic peptide reduces TNF-alpha-induced actin polymerization and endothelial permeability. Circ Res.

[41] Perkins ND (2012). The diverse and complex roles of NF-kappaB subunits in cancer. Nat Rev Cancer.

[42] Julien S, Puig I, Caretti E, Bonaventure J, Nelles L, van Roy F, Dargemont C, de Herreros AG, Bellacosa A, Larue L (2007). Activation of NF-kappaB by Akt upregulates Snail expression and induces epithelium mesenchyme transition. Oncogene.

[43] Tsubaki M, Komai M, Fujimoto S, Itoh T, Imano M, Sakamoto K, Shimaoka H, Takeda T, Ogawa N, Mashimo K, Fujiwara D, Mukai J, Sakaguchi K (2013). Activation of NF-kappaB by the RANKL/RANK system up-regulates snail and twist expressions and induces epithelial-to-mesenchymal transition in mammary tumor cell lines. J Exp Clin Cancer Res.

[44] Zhang Q, Helfand BT, Jang TL, Zhu LJ, Chen L, Yang XJ, Kozlowski J, Smith N, Kundu SD, Yang G, Raji AA, Javonovic B, Pins M (2009). Nuclear factor-kappaB-mediated transforming growth factor-beta-induced expression of vimentin is an independent predictor of biochemical recurrence after radical prostatectomy. Clin Cancer Res.

[45] Vermeulen L, De Wilde G, Van Damme P, Vanden Berghe W, Haegeman G (2003). Transcriptional activation of the NF-kappaB p65 subunit by mitogen- and stress-activated protein kinase-1 (MSK1). EMBO J.

[46] Saccani S, Pantano S, Natoli G (2002). p38-Dependent marking of inflammatory genes for increased NF-kappa B recruitment. Nat Immunol.

[47] Chew J, Biswas S, Shreeram S, Humaidi M, Wong ET, Dhillion MK, Teo H, Hazra A, Fang CC, Lopez-Collazo E, Bulavin DV, Tergaonkar V (2009). WIP1 phosphatase is a negative regulator of NF-kappaB signalling. Nat Cell Biol.

[48] Krubasik D, Iyer NG, English WR, Ahmed AA, Vias M, Roskelley C, Brenton JD, Caldas C, Murphy G (2006). Absence of p300 induces cellular phenotypic changes characteristic of epithelial to mesenchyme transition. Br J Cancer.

[49] Yokomizo C, Yamaguchi K, Itoh Y, Nishimura T, Umemura A, Minami M, Yasui K, Mitsuyoshi H, Fujii H, Tochiki N, Nakajima T, Okanoue T, Yoshikawa T (2011). High expression of p300 in HCC predicts shortened overall survival in association with enhanced epithelial mesenchymal transition of HCC cells. Cancer Lett.

[50] Brown JR, DuBois RN (2004). Cyclooxygenase as a target in lung cancer. Clin Cancer Res.

[51] Hida T, Yatabe Y, Achiwa H, Muramatsu H, Kozaki K, Nakamura S, Ogawa M, Mitsudomi T, Sugiura T, Takahashi T (1998). Increased expression of cyclooxygenase 2 occurs frequently in human lung cancers, specifically in adenocarcinomas. Cancer Res.

[52] Wolff H, Saukkonen K, Anttila S, Karjalainen A, Vainio H, Ristimaki A (1998). Expression of cyclooxygenase-2 in human lung carcinoma. Cancer Res.

[53] Tsujii M, Kawano S, DuBois RN (1997). Cyclooxygenase-2 expression in human colon cancer cells increases metastatic potential. Proc Natl Acad Sci U S A.

[54] Dohadwala M, Luo J, Zhu L, Lin Y, Dougherty GJ, Sharma S, Huang M, Pold M, Batra RK, Dubinett SM (2001). Non-small cell lung cancer cyclooxygenase-2-dependent invasion is mediated by CD44. J Biol Chem.

[55] Takai E, Tsukimoto M, Kojima S (2013). TGF-beta1 downregulates COX-2 expression leading to decrease of PGE2 production in human lung cancer A549 cells, which is involved in fibrotic response to TGF-beta1. PLoS One.

