# Supplementary material for: Inhibition of cancer cell epithelial mesenchymal transition by normal fibroblasts via production of 5-methoxytryptophan
Source: Oncotarget. 2016 Apr 29;7(21):31243–56. doi: 10.18632/oncotarget.9111 (PMC5058753; doi:10.18632/oncotarget.9111)
Supplement: Supplementary file 1 [file oncotarget-07-31243-s001.pdf]

# Inhibition of cancer cell epithelial mesenchymal transition by normal fibroblasts via production of 5-methoxytryptophan

## Supplementary Materials

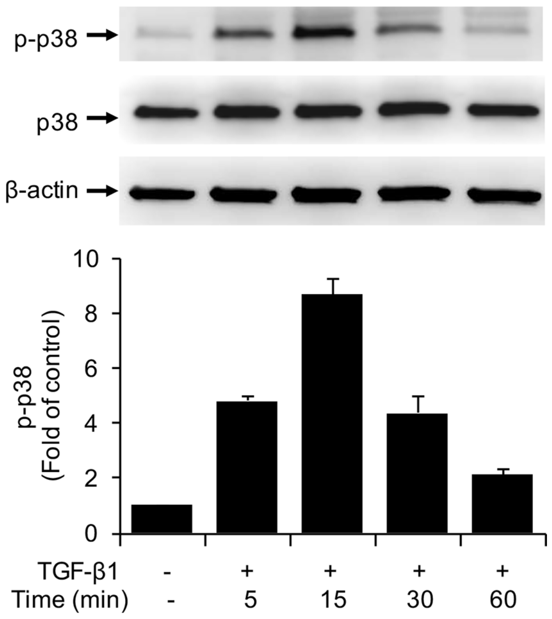


**Supplementary Figure S1: TGF-β1 activates p38 MAPK in a time-dependent manner.** A549 cells were treated with TGF-β1 (5 ng/ml) for indicated time. The upper panel shows representative Western blots and the lower panel the densitometry. Error bars denote mean ± SEM (*n* = 3).

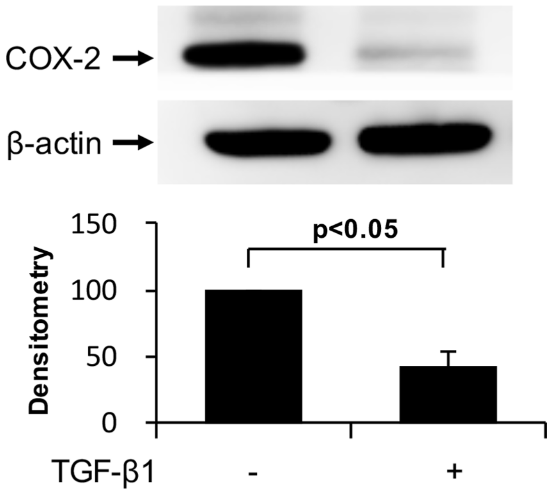


**Supplementary Figure S2: Suppression of A549 COX-2 expression by TGF-β1.** A549 cells were treated with TGF-β1 (5 ng/ml) for 48 h. COX-2 proteins in cell lysates were analyzed by Western blotting. The error bars indicate mean ± SEM (n = 3).

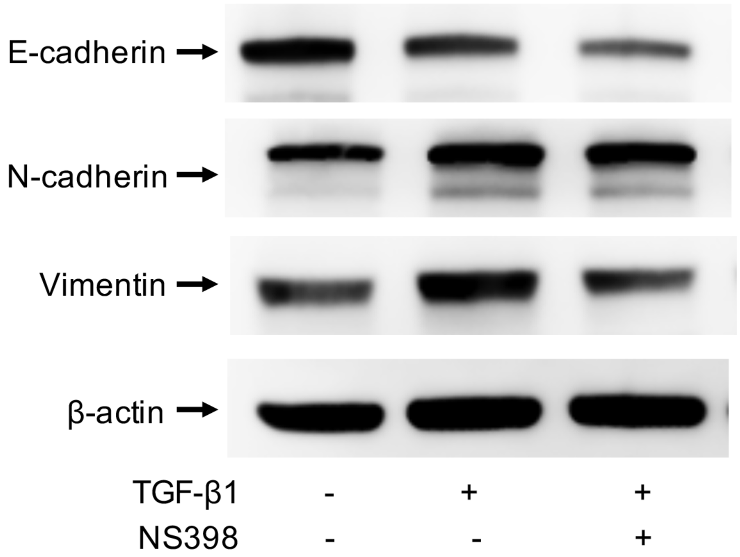


**Supplementary Figure S3: Selective COX-2 inhibition does not alter EMT.** A549 cells were pretreated with selective COX-2 inhibitor NS398 (10 μM) followed by treatment with TGF-β1 for 48 h. EMT markers in cell lysates were analyzed. This blot is representative of two experiments with similar results.
